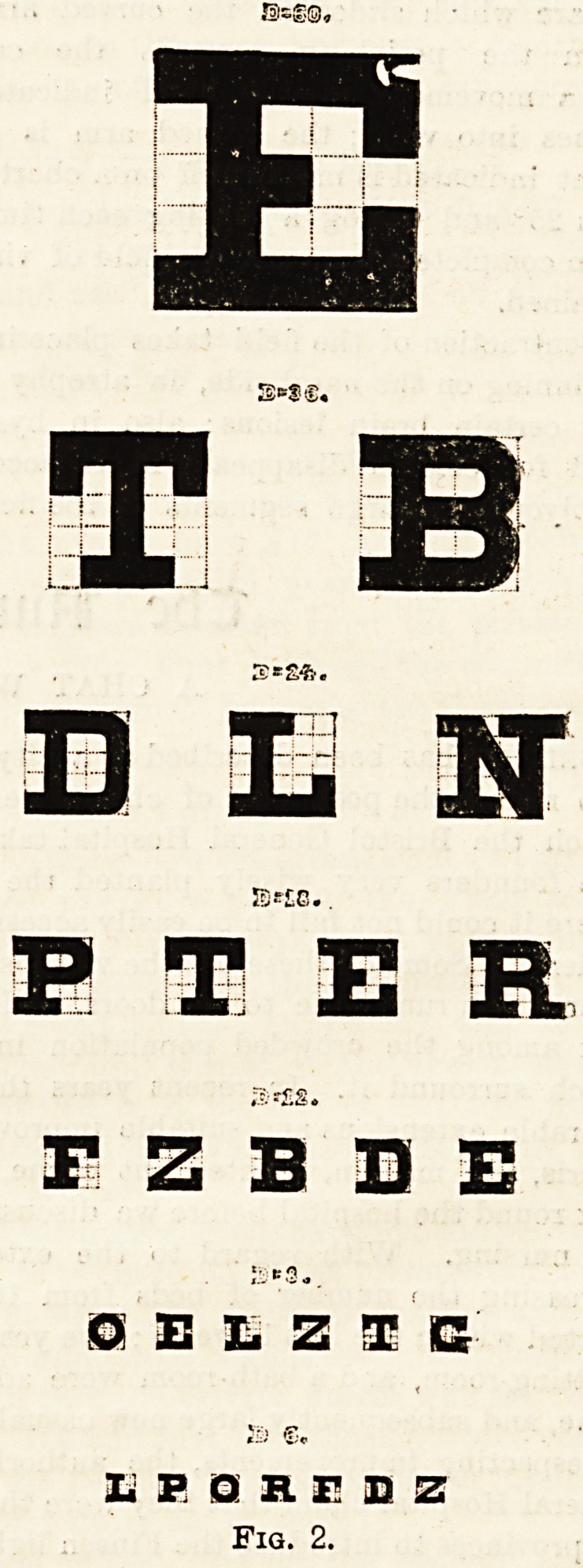# The Hospital. Nursing Section

**Published:** 1903-11-21

**Authors:** 


### The Hospital.

### Nursing Section.

Contributions for this Section of "The Hospital" should be addressed to the Editor, "The Hospital" Nursing Section, 28 & 29 Southampton Street, Strand, London, W.C

No. 895.—Vol. XXXV. *SATURDAY, NOVEMBER* 21, 1903.

### Notes on News from the Nursing World.

### THE KING OF ITALY AND THE ITALIAN HOSPITAL.

Although, owing to the shortness of their stay in London, the looked-for visit of the King and Queen of Italy in person to the Italian Hospital is not practicable, the sisters-in-charge have the satisfaction of knowing that both their Majesties take the deepest interest in their work. The nursing is entirely in the hands of the sisters of St. Vincent de Paul. They come to the hospital already trained, and are usually assisted by two or three secular nurses. The latter receive payment, but the work of the sisters is of course voluntary, uniform only being provided. The care of the theatre is in the hands of one of the sisters, and they also have charge of the dispensary and of the kitchen, where the preparation of Italian food is of great advantage to the patients, many of whom are from Italy. All nationalities are, however, admitted, including many English, and the sisters, of whom there are nine, speak both languages. Except in the case of the secular nurses, who have their fixed hours off duty, no regular leisure is arranged for, and the sisters are always astir at four o'clock in the morning and at work till late, two or three being on duty at night. Portraits of Queen Elena and Queen Margarita hang in the board-room. The magnificently bound volume of views of the wards, presented to their Majesties at the Italian Embassy by Madame Ortelli, hon. lady superintendent of the hospital, was prepared from photographs specially taken during the summer months.

### LORD ROBERTS AND HIS NURSES.

Although the illness of Lord Roberts is not, we are glad to say, of an alarming character, and he is making excellent progress, it was wisely decided as soon as it was found that the Commander-in-chief was suffering from pneumonia to call in two nurses. Both Lord and Lady Roberts attach the utmost importance to skilled nursing, and have had experience of its value in the case of one of their own daughters. A nurse has been in constant attendance upon the distinguished patient day and night, at Englemere, Ascot, ever since he was ordered to bed. Lord Roberts, it is said, would be a better patient if he could be persuaded not to think about the War Office during his enforced inactivity.

### BRISTOL GENERAL HOSPITAL AND THE PENSION FUND.

Four years ago the authorities of the Bristol General Hospital decided that all new sisters, most of whom are selected from the private nursing staff, must belong to the Royal National Pension Fund for Nurses. Our Commissioner, in an interview with the matron, which is reported in another page, inquired how the arrangement works out. The matron stated that the committee pay £6 a year to the Fund for each private nurse, and the nurse herself pays £6 a year. This being continued, she becomes entitled, at the age of 50, to a pension of £22 10s. a year from the Pension Fund, the committee of the hospital adding what they think proper according to the period of service. In addition to the matron, the assistant matron, and the night superintendent, four sisters, and upwards of twenty nurses of the private staff have joined the Pension Fund, and everyone at the Bristol General Hospital finds that the scheme works well. We commend the admirable arrangement in operation at that institution to the attention of other large provincial hospitals where the nurses do not at present enjoy the same advantages.

### THE REPLY OF THE AUTHORITIES OF DUDLEY HOSPITAL.

The reply of the authorities of the Guest Hospital at Dudley to the serious statements made in our columns last week respecting their hesitation to rectify an extraordinary mistake in the certificate presented to one of the nurses trained in that institution, is that "the committee, having treated the nurse with the utmost consideration, scarcely think that it would be in her interests to reopen the matter." This rejoinder, as will be seen by a letter from the secretary in another part of the paper, is all that the committee have to say; and, of course, it leaves untouched the facts with which we dealt and the criticisms which we considered it necessary to offer. It was not for the sake of the nurse only that we took up the question. There is a wider issue involved than that of the interests of any individual. If the nurse has done anything which, in the opinion of the committee, would justify the refusal of a certificate dated and signed correctly, it ought never to have been given her in an incorrect form. It now remains for the subscribers who are concerned for the credit of the training school to press for the reopening of a matter which the committee propose to regard as closed.

### A WARNING TO NURSING ASSOCIATIONS.

An action involving two issues of considerable interest to the nursing world was tried at the Manchester Assizes last week before Mr. Justice Jelf and a special jury. The plaintiffs, Mr. and Mrs. Hall, sued the Oldham Nursing Association for damages as compensation for personal injuries alleged to have been sustained by Mrs. Hall through negligence on the part of two nurses employed by the organisation. There was no question as to the injuries themselves, but the evidence as to the contributory cause was conflicting. The right leg and left foot of the patient were badly burned, in one instance to the bone, by the application of hot water bottles. For the plaintiffs it was contended that the injuries were due to the carelessness of the nurses, after an operation; and the defence was that they took place during the operation itself, and that the Association being a charity it was not responsible for the acts of its nurses sent out on the application of patients. Dr. Robertson, of Oldham, and two other doctors who assisted at the operation, said that hot-water bottles were not applied when Mrs. Hall was on the operating table, and their application later, when she was placed in bed, was at the nurses' discretion; but doctors on the other side, who were not present at the operation, expressed their belief that the injuries must have been inflicted whilst she was on the table. The jury preferred to accept the testimony of the doctors who were present, and having found that the injuries were the result of the negligence of the nurses, they held the Oldham Nursing Association liable for damages which they assessed at £300. There may be room for a difference of opinion as to when the injuries were sustained by Mrs. Hall; there is none as to the soundness of the conclusion that the Association is responsible for the actions of its nurses. We cannot understand how, in face of the evidence that the Committee reserve the power to withdraw a nurse at any moment, receive all the fees she earns, and pay her a regular salary, they were advised to disclaim their obligation. It is true that they pleaded that the Association is a voluntary philanthropic body not carried on for profit, but if their nurses are their servants when they attend the sick poor gratuitously, they are none the less so when they attend the well-to-do for payment. The case is an object-lesson of the risks of combining two branches of nursing which are better kept apart.

### NATIONAL MEMORIAL TO NURSING SISTERS.

With reference to the proposal by the Matrons' Council for the erection of a national memorial to the nursing sisters, who died while they were employed in South Africa in the service of their country, attention is called by the Honorary Secretaries of the Ladies' Committee of the Capetown Cathedral Memorial Fund to a fact of considerable interest bearing upon the matter. Just two years ago, a sum of £300, contributed by the nursing sisters in South Africa for the erection of windows in the memorial portion of the Cathedral in memory of those of their number who gave their lives in the performance of their duty during the war, was sent to the Fund. If there is to be a national memorial we think that the suggestion that it should be in keeping with this memorial of the nursing sisters in South Africa is an excellent one, and that any money subscribed could not be better employed than in devoting it towards the erection of that part of the fabric in which the windows will be placed. As it is intended to inscribe on the walls of the Cathedral the names of all who lost their lives in the war, irrespective of sex, rank or religion, it would be exceedingly appropriate that a portion of the sacred building should form a national memorial to the nurses whose names are recorded in the interior.

### NURSING AT LADYWELL WORKHOUSE.

We understand that the guardians of St. Olave's Union will, on Friday, come to a decision respecting the duties of the superintendent nurse of Ladywell Workhouse, as revised some time ago by the medical officer and the clerk to the guardians in consultation. We have had an opportunity of comparing the duties detailed in the new form with those set forth in the old form, and we notice several improvements, one being the addition of a rule requiring the superintendent nurse to report to the medical officer daily at his first visit in the book provided for that purpose a summary of the whole of the cases under nursing, and, where required, any detailed reference to any case and on any matter of which the medical officer should be informed, or his instructions taken. The revised form has been in print for some time; to the duties prescribed in it the superintendent nurse takes no exception; and there is no reason why it should not at once be adopted. We hope, however, that this reform is only preliminary to the introduction of a staff of trained nurses in Ladywell Workhouse. At present the superintendent nurse only is required to be fully trained, it being a mere incident that the assistant matron is also a trained nurse. Seeing that of the 700 odd inmates of the workhouse the majority average the age of 70, it is obvious that they want more nursing than can be given by untrained assistants and attendants. As we pointed out in September—when there was no superintendent in the building—if the assistant matron did not happen to be a trained nurse, there would be no one to take her place in the unavoidable absence of the superintendent. This has always been the case at Ladywell, but it is time that an extremely unsatisfactory state of things should be remedied by arrangements which will ensure the perpetual presence of more than one untrained nurse in the workhouse.

### GIRLS FROM SCHOOL AS PROBATIONERS.

It would have been better if at the annual meeting of the Association of Poor-law Unions, which took place last week in London, the delegates had summarily disposed of a declaration on the part of the council in favour of the minimum age qualification for probationers in workhouse infirmaries being fixed at 18. It appears, however, that the course pursued by the majority of delegates was to send back to the council their report as to the recommendations of the Departmental Committee on Nursing for reconsideration. It is very unlikely that the President of the Local Government Board, who has been advised by the Departmental Committee to fix the minimum age at 21, will entertain the proposition to make it 18. The theory of the council of the Association of Poor-law Unions is that if candidates cannot be accepted as probationers until they are 21 they will seek other means of employment. That may be so. But any possible difficulty in obtaining a sufficient number of probationers is infinitely preferable to entrusting what some of the lady delegates at the meeting called "girls from school" with the serious and arduous work of nursing the sick poor. It is curious that at a time when some of the workhouse infirmary-trained nurses are protesting in our columns against the best posts being given to their hospital-trained sisters, a minimum age which would still further damage their prospects should be advocated by responsible persons. Twenty-one is three years earlier than probationers are accepted at the leading general hospitals, and this is probably among the reasons why hospital-trained nurses so often subsequently carry off the "Poor-law prizes." We maintain that, whether in the interests of workhouse infirmary-trained nurses themselves or of the helpless patients committed to their care, the age minimum must not be reduced to 18.

### TRAGIC DEATH OF A NURSE.

On Monday morning a young woman attired as a nurse was found unconscious on the London and North-Western Railway, between Crewe and Sandbach, shockingly injured. She was conveyed to the Cottage Hospital at Crewe, and was identified as Miss S. Hodgkinson, a nurse at Uttoxeter Infirmary, and daughter of a veterinary surgeon in the town. The following letter, found in one of her pockets, dated from the Euston Hotel, London, suggests that she intended to commit suicide:—"For the inquest.—Sir,—Just a line to let you know I am taking my own life. I am not mad, only strange at times; but that is nothing; so you must do what you like to dispose of my body. Be as kind as you can with my mother and relatives. I have sent them all word, and I hope they will all soon forget me; but when you are ill, and no money, you don't like to sponge on your people or relations, although they have all been so kind in coming forward to help me in my ——, but I cannot go on for it. Fancy, six months. I was one for work, and have always been so strong.—Believe me to remain, yours sincerely, Nurse S. Hodgkinson." When she was examined at the hospital a plaster was found over her heart, and, on this being removed a photograph of a young man was discovered underneath. She died on Tuesday night without recovering consciousness.

### NURSES AND TOTAL ABSTINENCE.

In response to the invitation of Miss Richardson, lady superintendent of the London Temperance Hospital, a considerable number of nurses visited that institution on Saturday afternoon last. A vocal and instrumental programme, given by sisters and nurses of the hospital, was much enjoyed and appreciated by the visitors. After tea Miss S. E. Orme, a vice-president of the Total Abstinence League, delivered a short address, and welcomed to the League with particular pleasure three sisters and seven nurses of the London Temperance Hospital who had joined that day. Miss Arnott, a member of the League, expressed the thanks of all present to Miss Richardson for her kind reception. The visitors were then shown round the wards, which interested them exceedingly as affording substantial testimony of the efficacy of temperance with treatment of disease.

### COMPLIMENT TO A COURAGEOUS NURSE.

Miss Lottie Burgess, who is attached to the Private Nurses' Home at Huddersfield, has been presented by the Royal Humane Society with a certificate in recognition of her courageous conduct in saving the life of a patient from drowning in the sea at Blackpool last July. Miss Burgess was trained at Sculcoats Union Infirmary, Hull, and was afterwards charge nurse. She has also done district nursing for the Queen's Jubilee Home at Hull.

### RESIGNATION OF A MATRON.

We understand that the nurses of the Kettering and District General Hospital are greatly concerned at the resignation of the matron, who has always taken the greatest interest in their work. They hope that Miss Ward will enjoy a long earned rest from the duties she has discharged to the entire satisfaction of the staff.

### NURSE AND NURSEMAIDS.

In one of the leading London daily papers last week, there appeared under the heading of "Nurse's Religious Mania," a report of a case of suicide of a girl of nineteen. The event and the circumstances alike are most regrettable, but we refer to the matter in order to suggest that more care might be taken by the daily press in mentioning avocations. The heading in this instance is the more inexcusable because one of the witnesses at the inquest was accurately described in the report as "a trained nurse." The unfortunate girl, whose father thought that she was suffering from religious mania, was a nursemaid in service at Lexham Gardens, South Kensington, and had no more to do with nursing than her fellow servant the parlourmaid.

### PRESENTATION BY NURSES TO A LADY MAYORESS.

Just before the retirement of Mr. John Royle from the office of Lord Mayor of Manchester, the Lady Mayoress was the recipient of a graceful compliment on the part of matron and nursing staff of Ancoats Hospital. The presentation was made by Miss Chambers, the matron, who was accompanied to the Town Hall by some of the sisters and nurses, and consisted of a handsomely illuminated album, containing portraits of the nursing staff at the Ancoats Hospital, and an address expressing sincere thanks for the entertainment which was given by the Lady Mayoress at Heaton Park in July. Mrs. Royle, in reply, said that she was greatly pleased with the souvenir.

### A PROSPEROUS SCOTTISH ASSOCIATION.

The managers of the Thurso District Nursing Association were able to announce, through the chairman, at the annual meeting that they had a balance at the end of the year on the capital account of £194. The record of work done is equally satisfactory, 3,371 visits having been paid by the nurse to 75 cases. In the spring a superintendent from Edinburgh came to Thurso and expressed her gratification at the manner in which the work was done.

### SHORT ITEMS.

A pleasant little gathering of district and workhouse nurses was held at Selworthy, Somersetshire, on November 9th, when a paper was read by a sister of the Army Nursing Reserve on her experiences in South Africa.—A very successful entertainment was given to the in-patients of the Cancer Hospital, Fulham Road, on Thursday evening last week by the Socials Democratic Company. The farcical comedy entitled "The Strange Adventures of Miss Brown," by the late R. Buchanan and Chas. Marlow, was acted. A vote of thanks, proposed by the Secretary, Mr. F. W. Howell, was accorded with acclamation.

### Lectures on Ophthalmic Nursing.

By A. S. Cobbledick, M.D., B.S.Lond., Senior Clinical Assistant and late House-Surgeon and Registrar to the Royal Eye Hospital.

### LECTURE XXIII.—TREATMENT OF CATARACT (*cont.*). INSTRUMENTS USED IN DIAGNOSING DISEASES OF THE VITREOUS, RETINA, AND CHOROID.—SIGHT TESTING.

The after-treatment of operation cases must be careful, for the same methods are not applicable to all cases; *e.g.* some old people become very troublesome, and at times delirious, if both eyes are kept bound up, so that a pad must be left off in 24 or 36 hours in these cases.

Although it is well to keep the patient flat on the back for at least four days, aged people with feeble circulation are apt to develop trouble at the bases of the lungs—hypostatic congestion—with consequent cough and embarrassed heart action. This class of case should be propped up in bed with pillows after the second day. Old people with chronic bronchitis and cough should be allowed to sit up early, and be provided with a large pad of wool to hold over the operated eye when the cough comes on.

Fig. 1.

It is imperative to make the dressing next the lids comfortable and unirritating, otherwise the patients attempt to rearrange matters by getting their forefingers beneath the dressing.

After the fourth or fifth day the sound eye may be unbandaged.

During the first twelve hours subsequent to operation, tears may become pent up within the lids and give rise to considerable discomfort; if the nurse thinks this is the cause of irritation, she should raise the pad and guard and gently draw down the lower lid so as to let out the tears. The pad and bandage should be carefully reapplied.

No attempt should be made to obtain an action of the bowels before four days have elapsed from the date of operation. At the end of a week or ten days, according to the condition of the wound, the patient may be allowed up, and at the end of a fortnight the pad may be discarded if the scar looks sound.

In nearly all cataract-extraction cases in old people there is sufficient débris and capsule left to impair good vision, and a capsulotomy should be performed in about three months' time from the extraction.

Many successful extractions obtain excellent vision after the latter operation, with, of course, the aid of strong convex glasses, which must do the duty of a sound crystalline lens.

Fig. 2.

*Diseases of the Vitreous, Retina, and Choroid.*—These diseases give rise to very serious loss of vision in what appears to be, from all external appearances, a normal eye. The diagnosis of these troubles is made by means of the *ophthalmoscope.* On account of the practical experience necessary in examining the posterior portion of a diseased eye, only a brief outline will be here given of these diseases. Nurses must be acquainted with certain instruments and methods used in the diagnosis of these diseases. The perimeter (fig. 1) is used to test the field of vision. We are all aware of the fact that, when looking straight in front, we have a knowledge of what is going on at either side of us: the images formed are indistinct and lack clearness of outline, but they are sufficiently marked to attract our attention. This indistinctness is due to the rays of light

**LECTURES ON OPHTHALMIC NURSING**—*Continued.*

falling on the retina towards its periphery. The most acute visional part of the retina, as we saw in an early lecture, is the *yellow spot* or *macula*, which is situated to the outer side of the optic disc, and it is this part which is brought to bear on an object when we wish to see it as clearly as possible.

This complete range of vision is called the *field of vision*. McHardy's perimeter is one that is frequently in use. A portable perimeter has lately been devised by E. B. James. The examination of the field of vision is of considerable value in the diagnosis of certain diseases of the eye. Each eye is separately examined: the eye to be tested must be fixed on a central white spot. A small white square which slides on the curved arm is slowly drawn from the periphery towards the centre, the patient by a movement of the hand indicates the moment it comes into view; the curved arm is graduated and the point indicated is marked off on a chart. By revolving the arm 25° and taking a reading each time until a circle has been completed, the complete field of vision for that eye is obtained.

Contraction of the field takes place in chronic glaucoma, beginning on the nasal side, in atrophy of the optic nerve, and certain brain lesions; also in hysteria. The central field for colours disappears in tobacco and other toxic amblyopias. Large segments of the field or the whole of one-half of the field of vision may be wanting in certain affections of the optic tract.

This is a suitable place to say a few words on sight testing; it is an important branch of a hospital nurse's work in the out-patient department, and requires considerable practice to become expert.

The distant vision is tested by placing the patient 6 metres from Snellen's types (fig. 2) and finding what can be read with each eye separately, and without a lens.

The vision is recorded by a fraction—the 6 metres being the numerator and the number of the line seen the denominator. Thus, if the top letter only can be seen with the right eye it is expressed R.V. $\frac{6}{60}$; if the sixth line can be seen with the left eye it is expressed thus :—L.V. $\frac{6}{9}$, and so on. If no letter on the type can be seen the vision is expressed as $\frac{6}{\text{nil}}$; in such cases the patient should be taken nearer to the type, and if he can see the largest letter at 2 metres the vision = $\frac{2}{60}$. When the largest letter cannot be seen at 1 metre, the ability to count fingers must be examined and noted; if fingers cannot be counted at all, note whether there is any perception of light.

Further remarks on sight testing will be continued in the next lecture.

### The Nurses of Bristol General Hospital.

A CHAT WITH THE MATRON. BY OUR COMMISSIONER.

Bristol has been described as a city of churches. It is also rich in the possession of charitable institutions, among which the Bristol General Hospital takes a foremost place. The founders very wisely planted the building in a spot where it could not fail to be easily accessible to many of the patients. Some of these are the victims of accidents on the river which runs close to its doors, and more still are the sick among the crowded population in the poor parishes which surround it. In recent years there have been considerable extensions and suitable improvements, which Miss Morris, the matron, pointed out to me in the course of our look round the hospital before we discussed the question of the nursing. With regard to the extensions, one block, increasing the number of beds from 160 to 200, has been erected within the last 14 years; five years ago 20 bedrooms, a sitting-room, and a bath-room were added to the nurses home, and subsequently large new casualty-rooms.

Respecting improvements, the authorities of the Bristol General Hospital claim that they were the first institution in the provinces to introduce the Finsen light, the date of the introduction being November, 1901. As the matron showed me, there are four lamps, and two *x*-ray apparatus, the most modern appliances being, in fact, in use. The desire that everything should be up to date is shown likewise by the improvements in the surgical wards, all of which have been refloored, repainted, and refurnished, lavatories also being attached. Quite a feature in the wards are the large tables with glass tops, which not only form handsome centre-pieces, but are also immensely useful as receptacles for everything needed. These, I learnt, were designed by Dr. Cookson, the late house-surgeon, Sister Thomas, the senior sister, and the matron. The electric light has been installed throughout the buildings, and everywhere there is evidence of the determination to keep pace with the times.

On our return to the matron's room I asked Miss Morris how many nurses were on duty in the wards?

"Seventy-two," replied the matron. "Of these, nine are ward sisters, and one home sister, who teaches the nurses massage, and takes all the classes. The committee arrange for her to have three months' training for the purpose, and we find that in nursing patients with fractures, a knowledge of massage is very important. Then, in addition to the matron and assistant matron, there is a night superintendent. Including the private staff, there are from a hundred to a hundred and twelve nurses. Those employed in the hospital are all, but the sisters, in their first, second, or third year of training."

### Probationers and Midwifery.

"You have, I think, a majority of paying probationers?"

"Nearly all are paying probationers, but we admit two or three each year without payment, who receive a salary from the outset, and sign for four years. They are trained for three and in the fourth they go on to the private staff."

"How much do the others pay?"

"The premium is £25, and the paying probationers are free to leave at the end of three years. But they usually stay and take the midwifery course, and then join the private staff for a year."

"About the midwifery course, do you train in the hospital?"

"No, in the district. We have a midwife and she usually has three or four pupils. Our probationers are trained on the understanding that they go in for the midwifery course."

"I suppose that this is a comparatively recent innovation?"

"We started midwifery courses six years ago. The advantages are obvious, one being that the nurses get their midwifery training without having to pay fifteen guineas."

"If they remain beyond the fourth year on the private staff what salary do they receive?"

"The salary the first year on the private staff is £26, and it goes up to £44."

"What are your arrangements for staffing the wards?"

"The wards, as you noticed, vary in size, but in two, containing 34 beds, we have on duty during the day a sister, two probationers in their first year, an assistant nurse who is in her second year, and a staff nurse who is in her third year. At night in these wards there are an assistant nurse and a staff nurse on duty. This represents the average."

"You have a sister in charge of the theatre?"

"In consequence of the great number of operations, a sister and a staff nurse are always on duty in the theatre, and a probationer when operations are proceeding. The theatre is about to be renovated, and before long we hope to have a new one. It is urgently wanted."

### The General Training.

"How long has the training here been for three years?"

"Since 1893. When I was appointed in that year the training was for two years, each nurse serving a year on the private staff. I was trained at the General Hospital, Birmingham, under Miss Fisher, and I was afterwards sister in charge of the theatre for six years. Then I came to Bristol as night superintendent at the Royal Infirmary, coming here, at the end of 14 months, as night sister. After I had been night sister for three months, and assistant matron for three months, I was elected matron."

"I conclude that the usual theoretical training is given?"

"The probationers and assistant nurses attend the courses of lectures on obstetric nursing, anatomy, physiology, and surgical and medical nursing, which are given respectively by two of the surgeons and two of the physicians. They take rough notes at each lecture, and copy them out at the end of the week. The wording of the certificate is very simple. It is to the effect that the possessor has received three years' training in the medical, surgical, obstetrical, and children's wards, and that she has attended the courses of lectures. Gold and silver medals are also awarded."

### Certificates of Merit.

"On what conditions?"

"The gold and silver medals are only awarded to the two nurses who have respectively passed through their training to the entire satisfaction of the matron, sisters, and hon. staff, medical and surgical, and who are the two best nurses of their year. I look over all the reports of the sisters as to their work in the wards. The two at the top receive the gold and silver medal, but others who are very near are awarded a certificate of merit. This, of course, is quite apart from the certificate of training. The medals were first distributed three years ago."

### Duty and Off Duty.

"At what hour do the day nurses go into the wards?"

"Half-past seven. They breakfast at seven, and go to prayers in the chapel before entering the wards. With regard to other meals, there are two dinners. The sisters and some of the nurses dine at 12.30, and the remainder of the nurses at 1. The sisters have supper alone at 7.30, the probationers at 8, and the staff and assistant nurses at 9. Prayers are said at 8.30 every evening in the chapel."

"How about the night nurses?"

"They breakfast at 8.30 p.m. and go on duty at 9. They have a meal at 12 and another at 4 in the ward kitchen. In the morning they are out from 9.30 until 12. We make no difference between the diet of the day nurses and that of the night nurses.'

"Do you consider that the off-duty time is on a liberal scale?"

"It was considerably augmented a few years ago, and the probationers have since enjoyed much better health. During the present year we have only had 14 nurses temporarily indisposed, and but two warded, one for appendicitis and the other for a poisoned hand. The probationers are off duty alternately every morning from 10.30 until 12, or 2 until 4.30; every other Sunday from 4 until 9.45. The Sundays on which they are not out they attend service in the hospital chapel. They have one long day each month, a night off when I can arrange, and three weeks' holiday in the year."

"The assistant nurses and staff nurses," continued the matron, "have the same holidays, but the former have one evening in the week off, from 5 until 9.45; one morning and two afternoons; the latter getting two evenings in the week and the one morning. The sisters have a month's holiday. They are off duty every other evening from 5 till 9.45, if the cases permit. On the same condition, they have one morning and one afternoon in the week, and once a month they are away from Saturday afternoon until Monday morning at 9 o'clock."

### Night Duty and Salaries.

"For what period do the nurses take night duty?"

"Three months. The night nurses have every Sunday evening off duty, and two days and a night off at the end of their term. I have just arranged that the probationers shall not go on night duty at all; they have hitherto done so."

"What salaries do you give the hospital staff?"

"The probationers who pay do not receive any salary the first year, but the others have £12, and all receive £14 and £16 their second and third years respectively. The sisters, most of whom are selected from the private staff, receive from £35 to £40. Four years ago the committee decided that all new sisters must belong to the Royal National Pension Fund."

### Nurses and the Pension Fund.

"I should like to know how the arrangement works out."

"The committee pay £6 a year to the Fund for each private nurse, and she herself pays £6 a year. Thus, the first year she receives £36 a year, the committee pay for her £6 to the Pension Fund, and the second £38, and £6 to the Pension Fund. The result is that at the age of 50 each of them will be entitled to a pension of £22 10s. from the Fund, the committee adding what they think proper according to the time of service."

"How many of the nurses, then, have joined the Fund?"

"From twenty to twenty-four of the private staff, and four sisters. The night sister, the assistant matron, and myself also belong to the Fund. Of course, the probationers cannot afford to join, and only the private nurses who are receiving a salary of £34. We find that this scheme works very well, and we consider the Pension Fund a wonderful institution."

"Is uniform compulsory out of doors?"

"For the private nurses. But for the hospital staff only indoor uniform is compulsory, though if they wear outdoor it must be black. The private nurses wear brown cloaks and bonnets out of doors."

### The Private Nurses.

"Do the private nurses have a month's holiday?"

"Yes, and when they are not busy at other times, we allow them to go home. Generally speaking, they are very busy, and we sometimes have to refuse three or four nurses a day. We supply the country districts all round, and as far off as Minehead and Ilfracombe. In fact, our private nurses have gone to Coventry, Stafford, and Burton-on-Trent. Two of our surgeons are in practice at the latter place, and they like to have our nurses. Of course, we give the doctors who always come to us the preference, and the hospital itself has the first claim."

"What duty do they take in the hospital?"

"It is usually arranged for them to take either sister's or special duty. They do all the sisters' holiday duty, and that pays the hospital for the time when they are not engaged. No separate accounts are kept. But the hospital owes the private nursing staff more than the private nurses owe the hospital."

The Home.

"I notice that although all the nurses reside in the home, separate quarters are allotted to the private staff."

"They have one floor to themselves. The night nurses also have their own corridor, and, as you have seen, there is a separate bedroom for every nurse. There are two general sitting-rooms, and each of the sisters has a sitting-room to herself."

"The rooms appear to be furnished very prettily, and there is no sameness about them, except that all are well lighted."

"A different style was as far as possible observed in furnishing. The home, while shut off from the hospital, is quite a part of it. The assistant matron is in charge."

"Have you any difficulty in obtaining probationers?"

"On the contrary, we have about four or five applications a day. I suppose that out of every 34 applications we have to refuse 23. Some of the nurses stay a long time at the hospital. The assistant matron has been here nearly 25 years, and three of the sisters 17 years. Many of our old nurses are now in prominent positions. One is the matron of the Bristol Children's Hospital, another is matron of the Convalescent Home here which was opened by the Queen, and a third is matron of the Eastville Infirmary."

Recreation and Sick Cookery.

"Do the committee provide any recreation?"

"They supply the monthly magazines, and a certain number of books. But we should like to have a proper library. The Committee, who are very generous as well as good men of business, may perhaps assist. I have only just got a home sister, and until I had one, a library was impracticable. The nurses could pay a small amount. It is a great help to me to have a home sister. In my opinion, it is necessary that the housekeeper at a large hospital should be a trained nurse, as well as that she should thoroughly understand nursing."

"Are any cookery lessons given to the nurses?"

"We have had four or five courses, and I am thinking of reviving them. It is important that some of the nurses should have practical lessons in sick cookery."

"You have a very large out-patients' department?"

"Yes, the patients are so numerous that there are always a sister and five nurses on duty. A mortuary was opened a year ago. The nurses collected the money for the linen and worked it themselves."

The Private Hospital.

"We have also" said the matron, as I rose to go, "a private hospital in connection with the public one for the use of the men working at the Bristol Dock extension. It was opened a year ago in September, and all the expenses, which are considerable, are paid by Sir John Aird. There are eight beds and a large out-patients' department, with a sister and a staff of nurses always on duty. We change the nurses every three months. The private hospital is on the same modern lines as the Bristol General."

### Wants and Workers.

Bandages for the eye, some six inches by four, others smaller, taped ready for use, are offered gratuitously to hospitals by a lady. Address L. A. S., 37 Tasman Road, Clapham Road, S.W.

A visiting nurse would be glad of old linen or left-off underwear for poor patients. Nurse Downes, Fernlea, Northcote Road, Sidcup.

### Everybody's Opinion.

[Correspondence on all subjects is invited, but we cannot in any way be responsible for the opinions expressed by our correspondents. No communication can be entertained if the name and address of the correspondent are not given as a guarantee of good faith, but not necessarily for publication. All correspondents should write on one side of the paper only.]

### GROSS CARELESSNESS AT DUDLEY HOSPITAL.

Mr. Arthur Bird, Secretary of the Guest Hospital, Dudley, writes: Seeing in your issue of last week an article having reference to the action of the committee of the Guest Hospital with regard to one of its nurses, I beg to say that the committee, having treated her with the utmost consideration, scarcely think it would be in her interest to re-open the matter.

### ROYAL NATIONAL PENSION FUND FOR NURSES.

"One of the First Thousand" writes: Perhaps some of the nurses who have joined the Pension Fund might like to know the opinion of one of the "First Thousand" as to the quinquennial valuation. If you will allow me, I will tell them that I, for one, am perfectly satisfied and am most thankful that I joined the Pension Fund fifteen years ago. Why some nurses prefer to fritter away their salaries instead of taking advantage of all that has been done for them by Sir Henry Burdett and other friends of nurses I cannot conceive. It is, I suppose, because they just live for the present, and never think of the time which comes, all too soon, when older nurses cannot obtain work but have to be shelved for the younger ones. I have heard nurses boast, "I am going to enjoy myself and get a good fling now. Someone is sure to look after me when I am old and poor." If the younger nurses could only be made to realize what those who have done their fair share of work in their profession know, the policy-holders of the Fund would in no time be doubled. I am glad to have this opportunity of thanking our noble founder, who in the most disinterested way espoused the cause of nurses by starting the Royal National Pension Fund, and also of testifying my appreciation of the kindness and courtesy of Mr. Dick, who never wearies in explaining all the details of the Fund, and in answering nurses' questions. How he remembers us all in the way he does is a marvel!

### ASEPTIC FLOORING FOR OPERATING THEATRE.

The Hon. Sydney Holland writes: In reply to "S. L." I really can speak with the confidence founded on experience when I say that next to mosaic, and perhaps even before it, as mosaic invariably cracks, the best floor is an ordinary deal floor covered with a really good linoleum, pattern going right through it. At the London Hospital, during the rebuilding of our operating theatre, we had such a floor, and 3,000 major operations were done over say a space of 10 feet by 6 feet. When this temporary theatre was closed, Dr. Bulloch, our bacteriologist, tried in vain to get any culture from the linoleum. Care must be taken that the linoleum is not put down on new boards, or there is a danger of dry rot. It can be laid equally well on "S. L.'s" Portland cement floor or on any stone floor. At the Poplar Hospital it has been down on stone stairs and passages for 13 years, and in the latter shows no signs of wear at all. If anyone were to give me a million, which is a happiness I despair of, to spend on a hospital, I would, if I had my way, which is unlikely, put down in the wards no other floor than wood covered with linoleum. It is quiet, warm, easily cleaned, non-absorbent, and pleasant for patients and nurses to walk on, which cannot be said of any other floor. Cork linoleum must be avoided.

### POOR-LAW VACANCIES AND GENERAL HOSPITAL TRAINED NURSES.

"London" writes: As a Poor-law trained nurse I have for some time past been impressed with the fact which "Margaret" rightly calls the injustice of hospital-trained nurses securing the best appointments in Poor-law infirmaries. Surely it is for Boards of Guardians to help us in this matter. We who have gone through our Poor-law training know the difference it makes in the wards whether we have a hospital-trained sister or a Poor-law trained sister over us. The latter will help and direct you, the former requires you to wait on her.

"Eastward-Ho" writes: I am indeed glad that at last someone has brought to light the unfortunate position which Poor-law trained nurses so often hold. It is quite true, as "Margaret" states, that the best vacancies in Poor-law infirmaries are nearly always filled with nurses trained in general hospitals. We who have been trained at some of the largest and most up-to-date infirmaries in London are often put aside for a nurse trained, perhaps, at a small general hospital. It is not fair! Reason tells one that we Poor-law nurses must be more competent to take charge of Poor-law patients, who are more or less chronic, than nurses who are used to only acute cases. If this condition of things remain, it is obvious that the splendid training given now by some of the infirmaries will be discontinued. For what will be the use? And who do we expect will take the trouble to go in for such a training when probably no position is forthcoming except perhaps charge nurse or day sister. Of course there are undoubtedly the few exceptions when the Poor-law nurse rises to a good position, and this is often by promotion, but by all appearances these exceptions are few and far between. A short time ago, on inquiring of a Poor-law matron why she always advocated that the hospital trained should fill all positions higher than day sister? her reply—sensible in a way—was, "Think how much more a nurse trained at the large general hospitals can teach my pro's than a nurse from another infirmary." We are willing to admit truth in this statement, in most cases, but what is the use of all this extra knowledge if our future positions are little more than nothing?

"Four Midland Nurses" write: We are glad to see that "Margaret" has taken up the question whether all the best Poor-law appointments should be given to hospital-trained nurses. The Poor-law authorities do nurses a great injustice in this respect, neither is it any encouragement to the doctors and matrons under whom they train. Poor-law methods are so essentially different that hospital-trained nurses are often entirely at sea in the large and responsible wards of the new and thoroughly up-to-date Poor-law training schools. To particularise: the testing of urine, the giving of hypodermics, and the responsibility of important dressings which they are rarely permitted to undertake, to us is daily routine and the very groundwork of our training. Furthermore, qualification in midwifery is a necessity with us; here the injustice is even greater, since the Poor-law authorities often give hospital-trained nurses the opportunity to learn this valuable branch of nursing in exchange for their services. We fear that if these appointments continue to be made the Poor-law service will not attract educated and refined women for training. We heartily agree with all that "Margaret" says, and are glad that someone has had the courage to open the question.

### TRAVEL NOTES AND QUERIES.

By our Travel Correspondent.

Hotels and Pensions in Rome (E.C.).—Kindly give a pseudonym in future. In the Via Ludovisi is the Hotel-Pension Eden, terms about 7 to 8 francs per day. Then there is the Hotel-Pension Hassler Piazza S. Trinita de Monti, close to the Spanish steps, a little more expensive, but almost all hotel proprietors come down in price to those who stay any length of time. You must remember, however, that you pay for sun. In the Via Cavour is the Albergo Liguria-Vallini, considerably cheaper, from 6 francs. Near the centre of the city Hotel Capitole at the corner of the Piazza Venezia, terms from 7 francs. Then for real Pensions, Miss Hayden, 42 Piazza Poli, will take you from 6 francs. Pension Lermann, 62 Via Bon Compagni, terms 7 to 10 francs. Pension Union, 121 Piazza di Monte Citorio, from 6 francs. If you require a room looking to the sun you will find the terms raised, but perhaps that is not imperative if you are out most of the day.

### Appointments.

[No charge is made for announcements under this head, and we are always glad to receive, and publish, appointments. The information to insure accuracy should be sent from the nurses themselves, and we cannot undertake to correct official announcements which may happen to be inaccurate. It is essential that in all cases the school of training should be given.]

Billingsgate Mission Hospital. — Miss Florence C. Fell has been appointed superintendent of nurses; Miss Florence M. Brooks sister in charge of the men's surgical and accident ward, and Miss Maria Judd. sister in charge of the out-patient department. They were all trained at Tottenham General Hospital. Miss Fell and Miss Judd have had experience in private nursing, and Miss Judd holds the L O.S. certificate.

Plymouth Workhouse Infirmary.—Miss Sarah A. Lewis has been appointed night charge nurse. She belongs to the Meath Workhouse Nursing Association, and has recently completed her three years' training at the Crumpsall Infirmary.

Royal Hospital for Sick Children, Edinburgh.—Miss Mary L. Appleyard has been promoted from night sister to assistant matron, and Miss Rebecca Marston sister of a medical ward. Both were trained at St. Bartholomew's Hospital, London.

### Presentations.

Bridge School, Witham.—Miss Emily Baker, matron, who is leaving Bridge School, Witham, to take up similar duties at High Wood School, Brentwood, has been presented by the doctors, nurses, and domestic staff with a silver tea set, silver-mounted oak tray, and other small presents, as a token of the respect and esteem in which they held her.

Ascot Royal Victoria Home —Miss Ida Mackintosh, who has been appointed lady superintendent of The Elder Cottage Hospital and College Nurses' Training Home, Govan, Glasgow, has been presented by the committees of the Ascot Royal Victoria Home with a solid silver inkstand, by the medical staff with a silver-fitted dressing bag, by old patients and friends with a travelling clock, by the nurses with a silver pen and pencil and silver manicure set, and anonymously with a cheque for £50.

### Novelties for Nurses.

By Our Shopping Correspondent.

### WARM UNDERWEAR.

Messrs. Greensmith Downes, George Street, Edinburgh, have sent patterns of their beautiful materials used in the underwear which they sell. The garments are well fashioned and finished, and the prices, all moderate, vary according to quality. In the finer makes they use a mixture of Australian wool and Indian cashmere, and in the heavier, excellent Australian wool. Their Scotch wincey is a delightfully fine and soft texture suitable for night-dresses, blouses, and costumes. The first cold of winter has already turned our thoughts to warmer clothing, and now is the time to send for patterns before deciding or purchasing hastily the first garments which come to hand.

### Echoes from the Outside World.

### Movements of Royalty.

On Monday the King and Queen, with Princess Victoria and the Prince and Princess of Wales, travelled to London from Sandringham by special train. Their Majesties arrived at Buckingham Palace shortly before three o'clock, at which hour a Privy Council was held by appointment, lasting only ten minutes. In the evening the Royal Family, accompanied by the Duchess of Argyll, travelled to Windsor Castle in order to be in readiness to receive the King and Queen of Italy, the Princess of Wales, accompanied by Prince Alexander of Teck, proceeding a little later to Frogmore Lodge. On Tuesday afternoon the King and Queen arrived at Windsor Station in an open carriage drawn by four horses, and accompanied by the Duke of Connaught and Princess Victoria, and escorted by a detachment of Life Guards. Her Majesty wore a dress of heliotrope silk, with a toque to match. When the train conveying the King and Queen of Italy stopped at the station, a most cordial greeting passed between the two monarchs and their consorts, King Edward and King Victor Emanuel embracing each other. The Mayor and Corporation of Windsor presented an address, and the King of Italy expressed his thanks in English. Then the two monarchs, the Duke of Connaught and the Prince of Wales entered the first carriage, Queen Alexandra, Queen Elena and the Princess of Wales occupying the second. Large crowds cheered their Majesties on their way from the station to the Castle, the most demonstrative welcome coming from the Italian residents who were massed near the Guildhall. A family dinner party, commencing at 8.45, took place in the Oak Room in the evening.

### The Italian Royal Visit.

The King and Queen of Italy, accompanied by Signor Tittoni (the Minister of the Royal Household), three aides-de-camp, and a doctor left Pisa early on Sunday morning. Nice was passed at two in the afternoon, the Royal train going through Macon at half-past one on Tuesday morning, whilst Versailles was reached at nine o'clock. King Victor Emmanuel expressed a desire that his *incognito* might be preserved, so that no great ceremonial was prepared by the authorities. Owing too to the recent arrest of an anarchist no one but a few journalists and Arsenal employes were allowed inside the Arsenal, where the King and Queen embarked on board the *Victoria and Albert* at four o'clock. Neither the King nor the Queen, who was dressed in a light grey costume with a train trimmed with blue velvet, appeared the worse for their 36 hours' railway journey. In the evening their Majesties entertained to dinner on board the Royal yacht the Maritime Prefect and several distinguished officers and commanders of British vessels. Early on Tuesday morning the Royal yacht steamed out of Cherbourg Harbour and the Royal visitors were met in drenching rain at Portsmouth by the Prince of Wales on behalf of King Edward. There was a great naval display. Luncheon over the travellers disembarked, and after being presented with an address of welcome, got into the special train for Windsor amid another salute of big guns. They reached their destination about three o'clock. The rooms set apart at Windsor for the Italian King are the Vandyck Room, the Picture Gallery, and the Queen's Closet; and for Queen Elena the Rubens Room, the Council Chamber, and the King's Closet.

### King Victor Emmanuel and Queen Elena.

In his youth King Victor Emmanuel was exceedingly frail. His mother, herself one of the most cultured women in Italy, undertook his early education, and found the boy so eager to learn that it was difficult to get him to take much physical exercise. One day his father was struck by his look of ill-health, confiscated all his books, and insisted on his learning to shoot and yacht and harden himself. This wise policy had its effect. King Victor Emmanuel, although small in stature and light in build, is singularly wiry. He can sit for hours in the saddle without fatigue, and, if need be, without food. He entered the army at 18, without making any special mark, although he is a good soldier; but all the time he was quietly training for the future, and his initial speech, after his father's death, came as a revelation to his people. His brave and stirring words roused his hearers to enthusiasm, as they cried "The master has come," and he has lived up to the promise he then gave. Everywhere he has effected reforms. A story is told of him that, soon after his accession, he turned up one morning at eight o'clock at the office of the Household, and found two lacqueys lazily dusting. Seizing a duster from one, and removing the dust from a writing table, no clerk being yet in attendance, he sat down to write his letters. At half-past nine a clerk sauntered in. "What time are you supposed to begin work?" "At eight, Sire." "Ah! I see you have not enough to do. I must get rid of some of you.' And he was as good as his word. The King is one of the first numismatists in Europe, having over 20,000 coins, all from Italian mints, and is a splendid electrician. In 1896 he married the beautiful Princess Elene of Montenegro, "the daughter of a strong race," who has borne him two little girls, to whom both parents are devotedly attached. They have been brought up on strictly hygienic principles, and their limbs have never been tied up in the swaddling clothes common to royal and wealthy babies in Italy.

### The King of Denmark.

On Sunday the fortieth anniversary of the accession of the King of Denmark to the throne was celebrated. No deputations were received by his Majesty, but many congratulatory addresses were sent to him from the cities and towns of his kingdom. In the morning the King and all the Imperial royal personages assembled in Fredensborg attended divine service in the Chapel, and in the evening there was a dinner party at the castle. King Christian IX., who on April 8th celebrated his 85th birthday, is now the doyen of European Sovereigns, having been born in 1818. But in length of reign he is exceeded by Francis Joseph I, who has been Emperor of Austria since 1848, and was crowned King of Hungary in 1867. On the occasion of the 40th anniversary of his accession, King Christian has been appointed a General in the British Army. His commission is to bear the date of November 15th, 1903.

### Serious Accident to Lord Kitchener.

Some alarm was caused on Monday by the news that Lord Kitchener had met with a serious accident. He was riding on Sunday from a country house, called Wildflower Hall, to Simla. On the road there is a small tunnel, and as the horse passed through it took fright at a coolie, who was almost invisible in the darkness, and collided with the side of the tunnel, throwing Lord Kitchener's leg with great violence against it. Both bones of the leg were broken clean above the ankle. Lord Kitchener was unaccompanied, and as the coolie who saw the accident bolted, he lay in the tunnel for half an hour in great suffering until some natives, to whom the incident was reported, brought an empty rickshaw and carried the disabled general to Simla, a mile distant. Medical attendance was at once obtained and the leg was set. The patient had a good night, and is reported to be doing well, but all his engagements for the next few weeks have necessarily been cancelled.

### A Book and its Story.

NEW NOVEL BY THE AUTHOR OF "THE SHUTTERS OF SILENCE."*

Mr. Burgin's new novel is attractive and well written, and it will find appreciative readers among those who like a wholesome story of country life, with cleverly delineated characters and scenes in which the dialogue abounds in humour. Its only defect is the very common one of being too drawn-out. Condensed into three parts instead of being spun out into four, it would have been a more artistic and equally interesting composition. As it is, the little more becomes the just too much, and weakens the effect of the whole. Apart from this, the book is delightful. Younger readers will follow the story of the twin sisters, who give the title to the book, with interest, and by older ones the delineation of Marcus Pendragon's character, a bachelor scientist, living on his country estate, will be appreciated. Marcus had many interested neighbours, but the two people who had the most kindly and unaffected anxiety for his welfare were his old friend, Dr. Hawtrey, the village doctor, and Whipple, his elderly butler. To lure him into the uncertain haven of matrimony was a design that more than once had been attempted. But to this Marcus was oblivious, or indifferent. Wholly engrossed in scientific research and chemical experiments—leading to accidents which needed all the care of Whipple and the skill of Dr. Hawtrey to rescue him from—the fact of his position, with its claims, was the last thing to which he gave attention. "As lord of the manor his entire ignorance of, and indifference to, the duties of his high office grieved Hawtrey, who thought that Marcus, instead of giving himself up to inventing unspeakable things productive of equally unspeakable smells, ought to take his proper place in the county, marry, and go to church regularly. On the other hand Marcus seldom went to church, and never married; he owned a liking for all explosives, save domestic ones." Dr. Hawtrey was treating Marcus, now convalescent, for an injury to his leg, the effects of a recent experimental explosion, when the story opens. "The doctor gazed from beneath the shelter of rugged brows, at his delicately-featured friend. The dreamy experimenter with picric acid looked very much like a human stork, whose moulting plumage was stained with grease and chemicals. On the other hand the doctor was fat, forty, energetic, and argumentative on the subject of matrimony. After much pressure Marcus says on one occasion, "What has matrimony to do with it? How can being married help a man to think of the world to come?" "How? Oh, you know nothing—nothing at all. I'm surprised at your ignorance. A bachelor has no right to be comfortable. Do you think the animals would have gone quietly into the Ark if they hadn't been allowed to do so in couples?" "Well, I'm not going into the Ark." Dr. Hawtrey was anxious to emigrate to Canada and hoped to "settle" Marcus before he started. A wife who would look after him, might, in a measure, take his place, and prevent, perhaps, the recurrence of "blow-ups" which had become alarmingly frequent of late. Marcus had another near neighbour, Lady Fitzgibbon, a handsome widow, with an impossible son, a boy of twelve, who was the terror of her acquaintances. Lady Fitzgibbon had decided to replace her late husband. She was forty-three, and fancied that Marcus was the only man in the neighbourhood she would care to put in his place. Her friend, Miss Smythe, though only incidentally, is a particularly good character with her sharp tongue, and kindly heart. They are asked to luncheon with Marcus one day to see his beautiful collection of orchids. When the day comes, Marcus, entirely oblivious of the fact that they had accepted his invitation, is engrossed in his laboratory, deep in some noisome experiment, when Whipple knocks at the door. "You told me to remind you her ladyship's coming to lunch, sir." "Lunch—Ladyship—Lady who?" "Lady Fitzgibbing, sir; and the other—the thin lady." "But I'm just in the middle of an extraordinary combination. This acid is too dangerous to leave." "No, it ain't, sir," said Whipple, "it's 'armless as a baby. The doctor took away the acid and buried it." "Then it's like his impertinence. Why?" "He said he didn't want to bury you too, sir. The ladies will be here in half an hour," said Whipple, "and I've got to valet you. Your fingers is stained dreffle, and you've damaged your shirt cuffs." Marcus suffered himself to be led back to the house by the persevering Whipple. His new clothes looked very nice when he straightened himself up and forgot to stoop. Whipple put a red rose into his buttonhole, and stepped back to study the effect. "I was in two minds about a norchid, sir," he said, with the air of a man who has painted an Academy picture; "but a rose is more Hinglish." Marcus was finished off by Whipple just in time to receive the guests. After luncheon the round of the orchid houses was made, and Marcus, still lame from the injury to his leg, accepted Lady Fitzgibbon's arm. His leg being very painful, Marcus, in spite of a stick, was glad of the support of Lady Fitzgibbon's arm, although he wished she had not been so snappy with Miss Smythe. Lady Fitzgibbon divined his thoughts as they entered the orchid house. "'You musn't mind my being snappy,' she said, softly. 'When a woman hasn't a man to guide her she loses her sense of proportion. You ought to share all this beauty,'" said Lady Fitzgibbon, as she took up a long spray of blooms. "'Certainly, with whom?' asked the unsuspecting Marcus. 'I often send them down to the cottage hospital.' Lady Fitzgibbon's fine eyes gazed dreamily into the air. 'Cottage hospital? a wife would appreciate them more. . . . It is surely an inadequate return to Providence that you remain single. Sometimes I, too, am very lonely. Sometimes I feel that Pepworth requires a syndicate to manage him. A mother isn't adequate.'" Miss Smythe had remained discreetly in the background during the walk. She was found in the library reading when they returned to the house. "'Why your book's upside down!' declared Lady Fitzgibbon. I believe you've been asleep.'" "'Oh, no, I haven't been asleep. I always read books upside down to get the epigrams right.'" But Marcus, with all his eccentricities, was more astute than he was accredited with being. Lady Fitzgibbon did not appeal to him in the least. He cherished a very different ideal. Here is a sketch of her as she appeared to Marcus one evening when entering their drawing-room before dinner. "Marion Frere stood by the fireplace; the pearly shimmer of her dress irradiated a beautiful personality, and accentuated its dreamy sweetness. It was impossible to imagine those half-revealing, half-clinging folds draping anyone else; they conveyed an impression of slumbering opals . . . There was a suggestion of sadness in the beautiful eyes—eyes which had dreamily gazed forth from her lattice for thirty years without seeing a Sir Lancelot ride by." But he comes, and she marries Marcus. After a short married life she dies, leaving him with twin girls—"the ladies of the manor." He only survives her a few months, and the rest of the book has to do with the twins. It is this part of the story which will delight girl readers. Here we leave them, and commend the book heartily, for the good points already enumerated.

* "The Ladies of the Manor." By G. B. Burgin. (Grant Richards. 6s.)

### For Reading to the Sick.

"THAT WHERE THOU ART THY CHILD MAY BE."

COME to me in the evening shade,
Or earth's low communings will soon
Of Thy dear face eclipse the light,
And change the fairest day to night.

Come to me in the midnight hour
When sleep witholds her balmy power;
Let my lone spirit find its rest
Like John, upon my Saviour's breast.

Come to me through life's varied way
And when its pulses cease to play
Then, Saviour! bid me come to Thee
That where Thou art, Thy child may be.

*Anon.*

"Now there was leaning on the bosom of Jesus one of the disciples whom He loved."

Since these words were written, we have all been numbered among "the disciples whom Jesus loves." We have His word for it. "As the Father hath loved Me, I also have loved you. Abide in My love." We have His life and death in proof of it, for "Christ died for us all," and "greater love than this no man hath, that he lay down His life for His friend." Moreover, our past lives and experience tells us not only that our Lord loves us, but unfolds the extent and depth of that love.

Love calls for love, and true love shows itself in deeds. The sick feel this, and they say: "How I wish I could be up and working for God, working for others. I seem to do so little; others are occupied from morning till night, and have something to show for the days that pass: but I lie here on a bed of pain and others have to wait on me, and I effect nothing."

Turn your eyes to the scene of the last Supper. Next to our Lord is St. John, the beloved disciple, leaning on the bosom of his Master, a privilege not given to any other.

And you are there, too. That is precisely your place, and it will be your place till you are strong and well. Then you will leave it, and, like St. John, work more actively for our Lord. St. John was an apostle, yes, but a disciple first: and if it is the same with you, are you out of heart, nay, could you wish for a more honoured post? At present, like the disciple whom Jesus loved, your home is on the breast of our Lord. There rest your aching head and your heart that often feels the pinch of disappointment: it is a pillow where both may find relief: and it is where our Lord invites you to rest awhile.—*Anon.*

Jesu, on Thy Bosom leaning,
Something of Thy Love I see,
O that I could learn Thy meaning;
"I in you, and ye in Me."

Do I long for Thee to hear me?
Long that Thou should'st speak to me
Thou art always more than near me;
"Thou in me, and I in Thee."

When mine eyes are vigils keeping,
Watching, waiting, wearily,
Thou I know art never sleeping;
"Thou in me, and I in Thee."

*Vernon Hutton.*

### Notes and Queries.

FOR REGULATIONS SEE PAGE 21.

*Baths.*

(65) Will you kindly give me any information at you disposal regarding baths in Lincolnshire, or elsewhere, where gynæcological cases are treated, and also state the cost of same.—*Anxious.*

Perhaps the Secretary of the Baths at Woodhall Spa may be able to help you, if you write to him.

*Abroad.*

(66) Will you kindly give me the address of a nursing home in Durban, Natal, and also give me the names of the medical men in London who control nursing affairs in South Africa and Buenos Ayres?—*A. E.*

We do not give the addresses of private nursing homes. The Matron, Natal Government Hospital, Durban, might possibly send you particulars of private nursing in Natal, and the Matron, the British Hospital, 74 Calle Perdeiel, Buenos Ayres, might also help you. Nursing affairs in Natal and Buenos Ayres are not controlled by medical men in London.

*Mental Nurse.*

(67) Will you kindly tell me the shortest period in which a mental nurse can become qualified; and also if it is best to enter an asylum or to go through the hospital wards of a workhouse for training? What would the cost be?—*L. S.*

Your best plan is to enter an asylum and earn the Medico-Psychological Society's certificate. The course occupies three years, but a salary is paid from the first. Apply to the Registrar, the Medico-Psychological Association, 11 Chandos Street, Cavendish Square, W., for advice.

*Maternity.*

(68) Will you kindly let me know where I could obtain maternity training free? I want two or three months' training before I go out as a missionary nurse I am a trained nurse.—*C. C.*

There is no free maternity training, but the Clapham Maternity Hospital, Jeffreys Road, Clapham, S.W., offers special terms to trained nurses.

Will you kindly tell me how many cases a candidate for the London Obstetrical Society must nurse? In the certificate of attendance during the lying-in period the person certifying says "has attended 20 cases during the ten days following labour, and has been in personal charge of five of these cases." What is the difference between "has attended" and "has been in personal charge?"—*U. N.*

Write to the Secretary of the London Obstetrical Society, 20 Hanover Square, W.

*Abroad.*

(69) I should like to obtain a post in Port Elizabeth, Cape Colony. Can you tell me if there is any institution to which I might apply? I have a certificate and excellent testimonials.—*A. S.*

You might apply to the Colonial Nursing Association, Imperial Institute, S.W., and to the Matron, Provincial Hospital, Port Elizabeth, Cape Colony. In applying for information in such cases as these applicants should always enclose stamps for reply.

*Home.*

(70) Will you kindly tell me of a home or institution where a nurse aged 57 could be received. She is no longer able to work, but is quite able to wait on herself. Her savings are exhausted, and she does not belong to the Pension Fund.—*H.*

The only nurses' charity for which she is eligible is the Trained Nurses' Annuity Fund, for particulars of which apply the Hon. Secretary, 72 Cheapside, E.C.; but it would be worth while to search through a local list of charities.

**Standard Nursing Manuals.**

"The Nursing Profession: How and Where to Train." 2s. net; 2s. 4d. post free.

"Nursing: Its Theory and Practice." (Revised Edition). 3s. 6d. post free.

"Elementary Anatomy and Surgery for Nurses." By William McAdam Eccles, M.D.Lond., M.B. 2s. 6d. post free.

"Elementary Physiology for Nurses." By C. F. Marshall, M.D., B.Sc., F.R.C.S. 2s. post free.

"Ophthalmic Nursing." By Sydney Stephenson, M.B., F.R.C.S., 3s. 6d. net; 3s. 10d post free.

"Nursing in Diseases of the Throat, Nose, and Ear." By P. Macleod Yearsley, F.R.C.S.Eng., M.R.C.S. 2s. 6d. post free.

"Fevers and Infectious Diseases." 1s. post free.

## Figures and Tables

**Fig. 1. f1:**
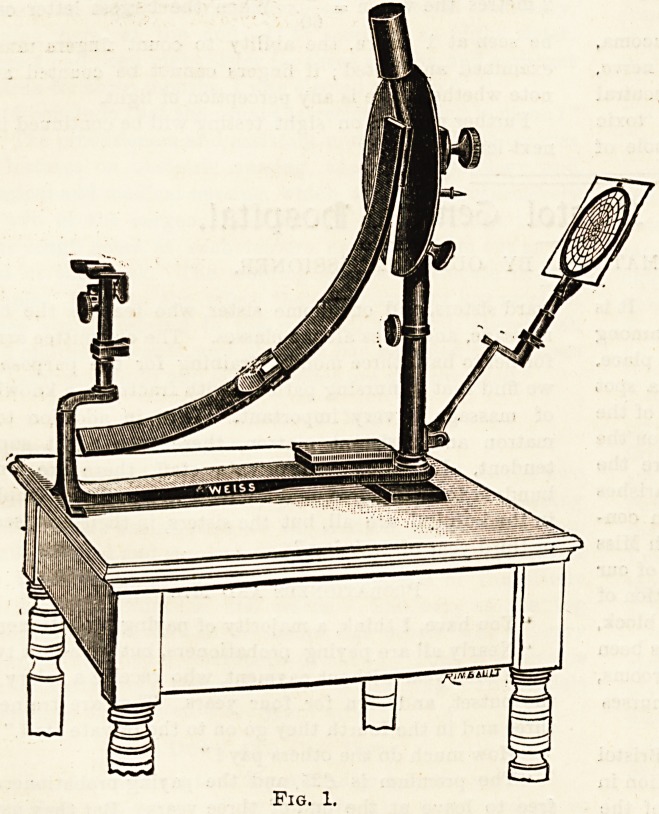


**Fig. 2. f2:**